# Effects of Black Raspberries and Their Ellagic Acid and Anthocyanin Constituents on Taxane Chemotherapy of Castration-Resistant Prostate Cancer Cells

**DOI:** 10.1038/s41598-019-39589-1

**Published:** 2019-03-13

**Authors:** Jillian N. Eskra, Michael J. Schlicht, Maarten C. Bosland

**Affiliations:** 1 Department of Pathology, College of Medicine, University of Illinois at Chicago, Chicago, IL USA; 2 Present Address: Department of Urology, Johns Hopkins University, Baltimore, MD USA

## Abstract

Cancer patients often use dietary supplements while on therapy, but little is known about interactions of supplements with cancer chemotherapy. Black raspberries (BRB) have anti-cancer effects, but have not been evaluated for interference with chemotherapy for castrate-resistant prostate cancer (CRPC). Here we studied whether BRB and some of their constituents interact with docetaxel and cabazitaxel on CRPC cells in culture and implanted into nude mice. Ellagic acid increased, but BRB extract inhibited, microtubule assembly. Ellagic acid decreased tubulin polymerization by cabazitaxel and bound to tubulin. Ellagic acid, its metabolite urolithin A, BRB extract, and the anthocyanin metabolite protocatechuic acid (PCA) did not alter cytotoxicity of taxanes. Ellagic acid inhibited drug efflux in CRPC cells, but BRB extract and PCA did not. None of these compounds altered CYP3A4 activity. Although dietary ellagic acid did not alter the tumor growth inhibition by docetaxel of xenografted 22Rv1 cells, ellagic acid has the potential to interfere with taxane chemotherapy by reducing tubulin polymerization while inhibiting P-glycoprotein drug efflux. These data are cause for concern of consuming ellagic acid during treatment for CRPC and indicate need for further research, but BRB consumption appears safe.

## Introduction

Castration-resistant prostate cancer (CRPC) is the lethal form of this malignancy that develops after androgen ablation therapy fails. Although treatment of CRPC is currently undergoing changes, it is usually still chemotherapy with a taxane drug, docetaxel being the first-line treatment and cabazitaxel a potential second-line option^1,2^. The problem with these chemotherapeutic approaches is the development of drug resistance. In addition to lacking substantial efficacy, increasing overall survival time by only a few months, it also tends to lead to severe impairment of patient quality-of-life^2,3^. Perhaps as a consequence, many men with prostate cancer, particularly those with more advanced stage disease, make dietary modifications or use some form of dietary supplements in addition to their standard of care therapy^4–7^. Despite the frequent use of supplements by cancer patients, little information exists on potential beneficial or harmful interactions between most supplements and chemotherapy drugs.

Black raspberries (BRB) have gained much attention as potential cancer prevention agents and BRB preparations are currently being investigated in several clinical trials^8^. While these trials focus on local effects of BRB on upper areodigestive and gastrointestinal tract^9,10^, there is experimental evidence indicating inhibitory effects of orally administered BRB on induction of mammary gland carcinomas in rats^11,12^. BRB contain many bioactive phytochemicals with known antioxidant and anticancer activity, inhibiting cell proliferation, inflammation, and angiogenesis and inducing apoptosis, cell differentiation, and adhesion. Their anti-cancer effects are mostly attributed to the high concentration of ellagic acid and anthocyanins^13–17^. In addition to preventive and therapeutic effects, BRBs could potentially be used as adjuvants to chemotherapy to enhance its effectiveness. However, there are no studies that have investigated the use of BRB for this purpose. Because many of the biological activities of BRB target similar pathways as do chemotherapeutic drugs, it is a plausible that adding BRB supplementation to chemotherapy could result in enhanced drug efficacy and reduced resistance.

Here, we investigated the ability of BRBs to modulate effects of taxane chemotherapeutics used in the treatment of CRPC. We hypothesized that treatment of prostate cancer cells with BRB extract and BRB compounds would improve effectiveness of the standard chemotherapeutics docetaxel and cabazitaxel, resulting in decreased growth of CRPC cells and increased sensitivity of these cells to chemotherapeutic agents. A secondary objective was to rule out possible adverse interactions, i.e., reduction in cytotoxic efficacy of docetaxel and cabazitaxel by BRB that may lead to harmful effects for patients who are consuming such supplements while on chemotherapy. We evaluated the effects on CRPC cells of combining docetaxel and cabazitaxel with BRB extract, ellagic acid and its metabolite urolithin A, and protocatechuic acid (PCA) which is a major metabolite of BRB anthocyanins.

## Results

### Ellagic acid increases but BRB extract inhibits microtubule assembly *in vitro*, but both decrease tubulin polymerization by cabazitaxel

**Figure 1:**
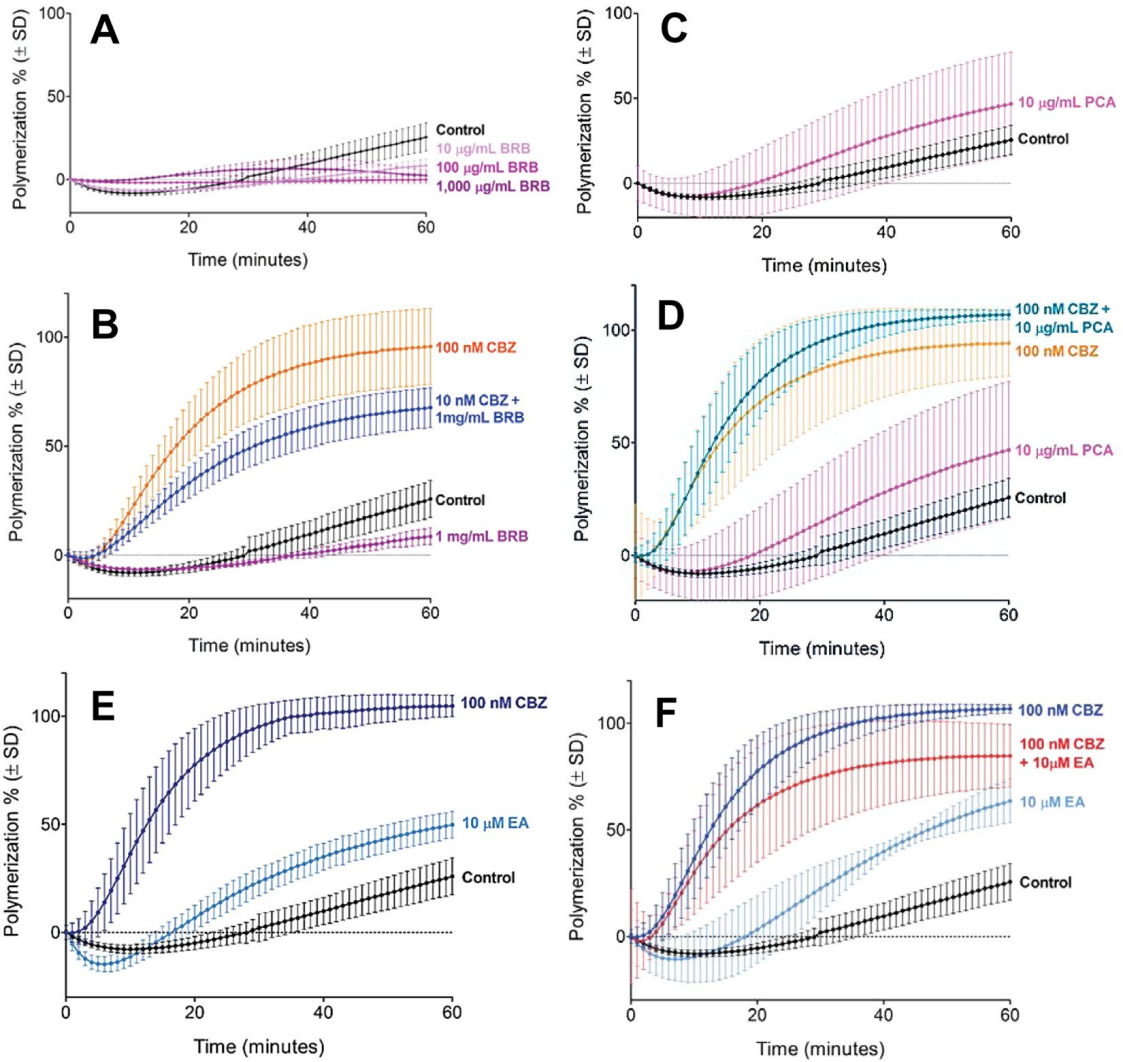
Effects of black raspberry extract (BRB), protocatechuic acid (PCA), ellagic acid (EA), and cabazitaxel (CBZ) on microtubule assembly *in vitro*. Tubulin polymerization was measured by fluorescence intensity at 60 minutes of exposure to 10–1,000 μg/mL black raspberry extract (**A**), 10 μg/mL protocatechuic acid (**C**), and 10 μM ellagic acid and 100 nM cabazitaxel (**E**), or combinations of 100 nM cabazitaxel with 1 mg/mL black raspberry extract (**B**), 10 μg/mL protocatechuic acid (**D**), and 10 μM ellagic acid (**F**). Data are mean values ± SD of 3–7 repeat measurements at 1 minute intervals.

We investigated the effects of BRB extract, PCA, ellagic acid, and urolithin A on tubulin polymerization using an cell-free assay. Tubulin polymerization was slightly reduced by BRB extract at concentrations of 10 μg/mL and higher (Fig. 1A) By contrast, 10 μM ellagic acid significantly increased the amount of polymerized tubulin compared to control (Fig. 1E), as did 10 μg/mL PCA although this effect was not significant (Fig. 1C). Urolithin A also induced tubulin polymerization, but this effect was minimal and not statistically significant (data not shown).

The combination of BRB extract (1 mg/mL) and 10 nM cabazitaxel decreased tubulin polymerization compared to the expected induction of tubulin polymerization by cabazitaxel alone (Fig. 1B). By contrast, 10 μg/mL PCA in combination with cabazitaxel resulted in a non-significant increase in tubulin polymerization over cabazitaxel treatment alone (Fig. 1D). The addition of 10 μM ellagic acid to cabazitaxel treatment (100 nM) resulted in a reduction of tubulin polymerization by about 20%, rather than the expected increase (Fig. 1F).

The significant *in vitro* effects of ellagic acid in the cell free microtubule polymerization assay were confirmed by assessment of microtubule assembly in 22Rv1 cells, which are capable of androgen independent growth and resemble the aggressive clinical phenotype of CRPC. Treating 22Rv1 cells with ellagic acid for 24 hours resulted in a dose-dependent increase in polymerized β-tubulin (Supplemental Fig. 1A).

**Figure 2:**
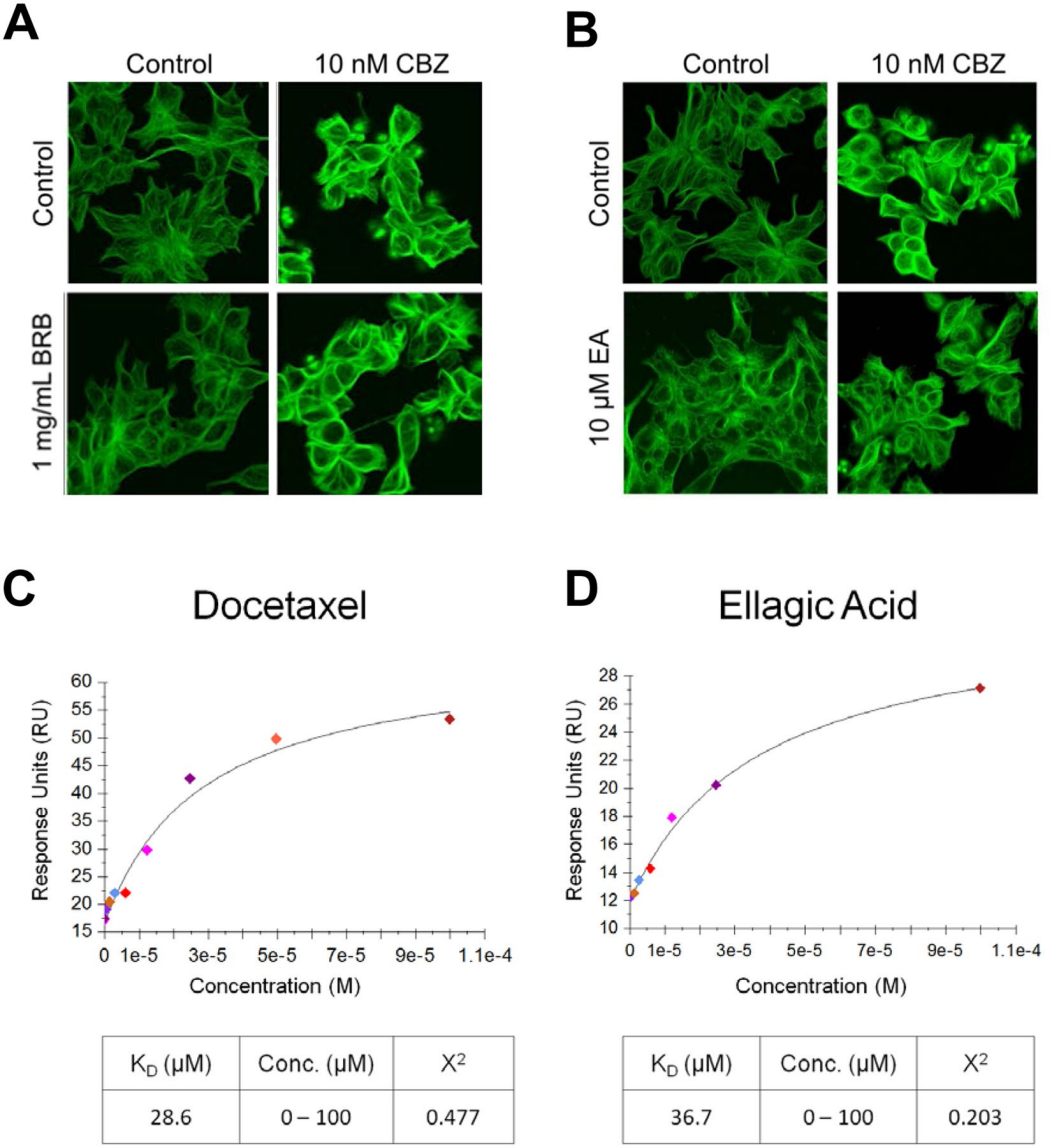
Combination effects of black raspberry extract (**A**) or ellagic acid (**B**) and CBZ on tubulin polymerization in 22Rv1 cells. Microtubules (green) were visualized by confocal microscopy with images enhanced for brightness and contrast. Binding affinity of docetaxel (**C**) and ellagic acid (**D**) for tubulin. This was determined by surface plasmon resonance and response-concentration plots are shown.

We further investigated the effects of BRB extract and ellagic acid in combination with cabazitaxel on microtubule assembly in 22Rv1 cells by confocal microscopy following 24 hour incubation with vehicle, 10 μM ellagic acid or BRB extract (1 mg/mL), 10 nM cabazitaxel, or 10 μM ellagic acid or 1 mg/mL BRB extract +10 nM cabazitaxel. Treatment with cabazitaxel alone caused a profound change in microtubule appearance visualized by confocal microscopy as an increase in microtubule density. BRB alone had no effect and adding BRB treatment to cabazitaxel did not change microtubule morphology in 22Rv1 cells compared to cabazitaxel treatment alone (Fig. 2A). By contrast, treatment with ellagic acid alone increased tubulin polymerization, while co-treatment with ellagic acid and cabazitaxel moderately decreased tubulin polymerization induced by cabazitaxel alone (Fig. 2B). Treatment with cabazitaxel also induced tubulin polymerization in western blot analysis, while co-treatment with BRB extract did not change this effect (Supplemental Fig. 1B). By contrast, treatment with ellagic acid moderately increased tubulin polymerization in western blot analysis while co-treatment with ellagic acid moderately decreased tubulin polymerization induced by cabazitaxel (Supplemental Fig. 1C).

### Ellagic acid binds to tubulin

The inhibition of microtubule polymerizing activity of taxanes by ellagic acid in a cell-free assay led us to speculate that these compounds may act directly on microtubules by binding to tubulin. We investigated potential binding interactions between ellagic acid and tubulin using a label-free, surface plasmon resonance assay. Docetaxel displayed high binding affinity for tubulin (Fig. 2C), while ellagic acid bound tubulin with lower affinity (Fig. 2D).

### Ellagic acid, urolithin A, BRB extract and PCA do not alter cytotoxicity of taxanes

**Figure 3:**
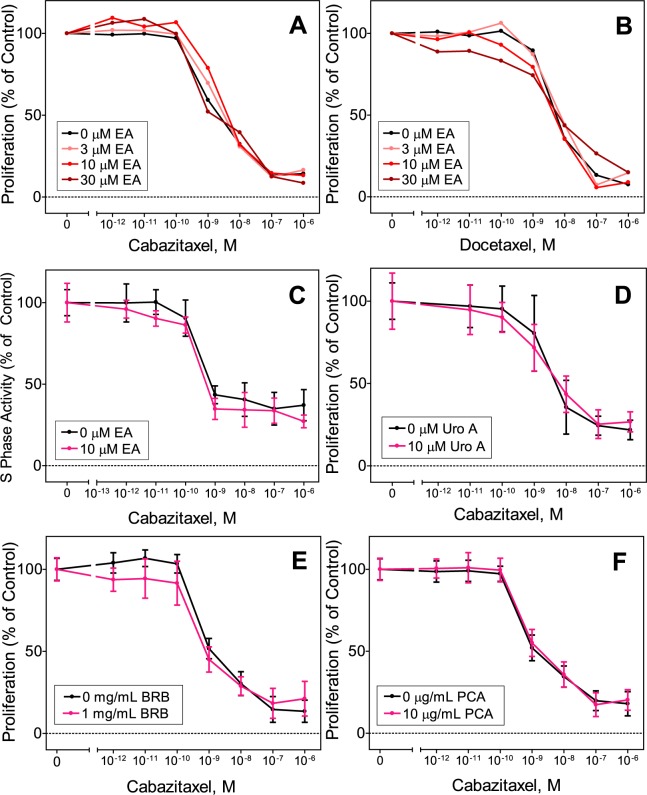
Combination effects on proliferation of 22Rv1 prostate cancer cells measured using the SRB assay after 72 hours treatment of 3–30 μM ellagic acid (EA) and 0.01–10 nM cabazitaxel (**A**) or docetaxel (**B**); combination effects of and 0.001–1,000 nM cabazitaxel and 10 μM urolithin A (Uro A) (**D**), 1 mg/ml black raspberry extract (BRB) (**E**) or 10 μM protocatechuic acid (**F**); and combination effects on S phase activity of C4-2 cells of 10 μM ellagic acid (EA) and 0.01–10 nM cabazitaxel (**C**) measured by incorporation of a thymidine analog into DNA. Results were normalized to vehicle control treated cells and are presented as mean ± SD of 3 independent measurements; for panels A and B the error bars are left out to make the curves shown easier to see; these panels are included with error bars in the Supplementary Material.

**Figure 4:**
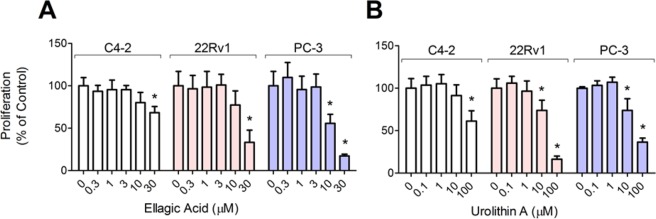
Effects of ellagic acid and urolithin A on proliferation of prostate cancer cells. Proliferation was measured in C4-2, 22Rv1 and PC-3 prostate cancer cell lines using the SRB assay after 72 hours treatment with 0.3–30 μM ellagic acid (**A**) and 0.1–100 μM urolithin A (**B**). Data normalized to vehicle control treated cells are presented as mean ± SD. *p < 0.05 for difference with vehicle control.

We hypothesized that the cytotoxicity of taxanes would be enhanced by co-treatment with ellagic acid and urolithin A, based on previously reported growth inhibitory effects of these two compounds on cancer cells^18,19^. However, results from our tubulin polymerization assays suggested that ellagic acid may reduce taxane efficacy by interfering with ability to induce microtubule polymerization. To examine this, we treated 22Rv1 cells with taxanes alone and in combination with ellagic acid or urolithin A. Proliferation of 22Rv1 cells treated for 72 hours with cabazitaxel and docetaxel in combination with ellagic acid did not differ significantly from cells treated with these taxanes alone (Fig. 3A,B). Likewise, 22Rv1 cell proliferation following combination treatment with urolithin A (10 μM), PCA (10 μg/mL), or BRB extract (1 mg/mL) was not different from cabazitaxel alone (Fig. 3D–F). Consistent with these results, S-phase activity (incorporation of a thymidine analog) in C4-2 cells treated with 10 μM ellagic acid in combination with cabazitaxel was not different from cells treated with the taxane alone (Fig. 3C). Ellagic acid and urolithin A by themselves reduced growth of C4-2 cells at concentrations greater than 10 μM and of 22Rv1 and PC-3 cells at concentrations of 10 μM and above (Fig. 4); BRB extract and PCA did not affect growth of these cell lines (data not shown). Taken together, these results suggest that BRB and none of the BRB compounds we tested altered the growth inhibitory effects of taxanes on CRPC cells.

### Ellagic acid inhibits efflux activity in 22Rv1 cells, but BRB extract and PCA do not

**Figure 5:**
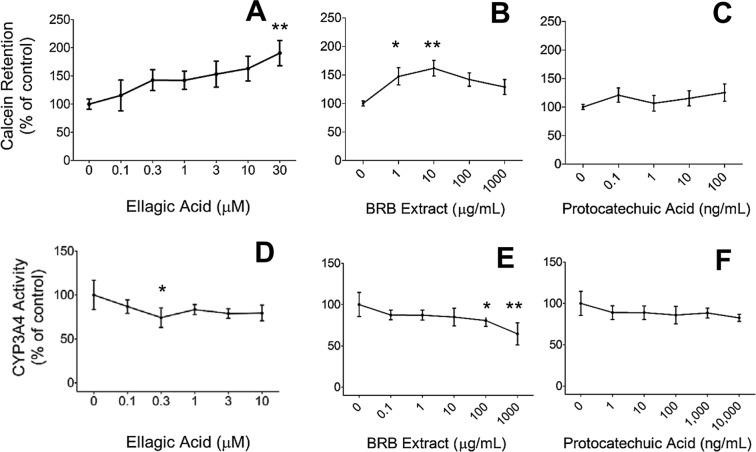
Effects on P-glycoprotein efflux activity in 22Rv1 cells of 0.1–30 μM ellagic acid (EA) (**A**), 1–1,000 μg/ml black raspberry extract (BRB) (**B**) or 0.1–100 μg/ml protocatechuic acid (**C**); and effects on CYP3A4 activity in CYP3A4 expressing microsomes of 0.1–10 μM ellagic acid (**D**), 0.1–1,000 μg/ml black raspberry extract (**E**) or 1–10,000 ng/ml protocatechuic acid (**F**). Data are presented as mean ± SD of 3–8 measurements. *p < 0.05 and **p < 0.01 for differences with vehicle control.

Increased activity and expression of ATP binding cassette (ABC) transporters, including P-glycoprotein, has been implicated in the development of resistance to numerous drugs^20,21^. The efflux activity of these proteins, which reduces intracellular drug accumulation, has been implicated in taxane resistance in CRPC patients^22,23^. Ellagic acid has been shown to inhibit activity of ABCG2, a drug transporter involved in drug resistance to chemotherapeutic agents, in ABCG2-overexpressing HEK293 cells^24^. Therefore, we examined the ability of ellagic acid (0.1–30 μM) to modulate efflux activity in 22Rv1 cells by measuring intracellular accumulation of the fluorescent dye calcein AM. We observed a dose-dependent increase in intracellular calcein (Fig. 5A), indicating a significant reduction in efflux activity by ellagic acid.

Effects of BRBs on activity of efflux transporters has not been investigated before, but P-glycoprotein inhibition by numerous dietary agents has been reported^25^. We evaluated efflux activity in 22Rv1 cells treated with BRB and PCA with the calcein AM method. We found no differences in intracellular calcein levels in cells treated with PCA compared to control, but observed a modest biphasic increase due to BRB extract with a maximum at 10 μg/mL (Fig. 5B,C), indicating BRB extract, but not PCA, may slightly reduce efflux activity.

### Ellagic acid, BRB extract and PCA do not alter CYP3A4 activity

CYP3A4 is the enzyme responsible for catabolizing taxanes^26^. CYP3A4 activity can be modulated by pharmaceutical agents, certain foods, and dietary supplements, including several polyphenolic compounds (28), which can have consequences for drug toxicity^27^. To investigate effects of BRB compounds on the catalytic activity of CYP3A4, we treated CYP3A4 expressing microsomes with ellagic acid, BRB extract and PCA, and then quantified activity by measuring the intensity of a fluorescence reporter metabolite of a CYP3A4 substrate. We did not observe an effect on CYP3A4 activity of ellagic acid and PCA at any concentration. However, we found a significant 20 and 36% reduction in activity at 100 and 1,000 μg/mL BRB extract, respectively (Fig. 5E), indicative of diminished taxane inactivation.

### Dietary ellagic acid does not alter efficacy of docetaxel on 22Rv1 tumor growth in mice

Because we observed significant effects of ellagic acid on tubulin polymerization and on the *in vitro* effects of taxanes, we examined the possible interaction of dietary ellagic acid with docetaxel treatment of subcutaneously xenografted 22Rv1 cells by assessing effects on tumor growth *in vivo* in nude mice. When tumors reached an approximate volume of 500 mm^3^, animals were placed on either control diets or diets containing 2 or 4 g/kg ellagic acid for the duration of the study. After 1 week of dietary intervention, mice received 3 injections of docetaxel (15 mg/kg) administered at 3 day intervals. Tumors were measured three times per week until tumor volume exceeded 3,000 mm^3^ or study termination 37 days after start of dietary intervention. We observed considerable variability in tumor volumes prior to chemotherapy and dietary interventions. Tumors that were not palpable (volume of at least 350 mm^3^) or had not reached the exponential growth phase at the start of docetaxel treatment were excluded from data analysis. Additionally, we observed three tumors (one each in the docetaxel + control diet, docetaxel + low dose ellagic acid, and docetaxel + high dose ellagic acid groups) with tumor volumes that exceeded 2,500 mm^3^ at the time of first docetaxel injection (day 35); these tumors were identified as outliers using Grubbs’ test and were also omitted from analysis.

**Figure 6:**
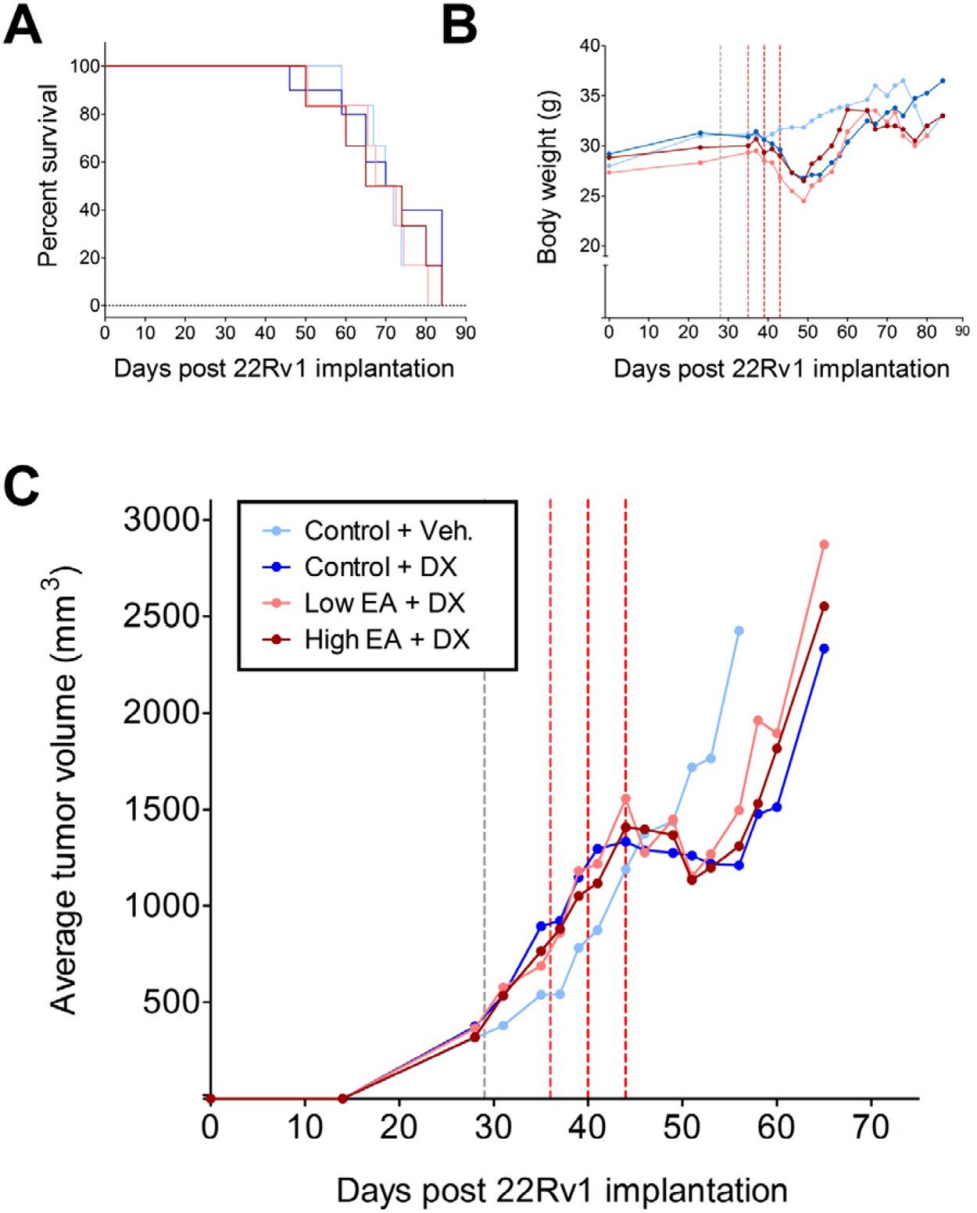
Ellagic acid does not enhance efficacy of docetaxel on 22Rv1 tumor growth in nude mice implanted with 22Rv1 cells in matrigel on day 0. Mice were switched from AIN93M diet to modified diets containing 2 or 4 g/kg ellagic acid on day 28 (gray dashed line) or maintained on the AIN93M control diet and received docetaxel injections on days 35, 39 and 43 (red dashed lines). Shown are survival curves (**A**), body weight (**B**), and average tumor volume (**C**). Details of these data are presented in Supplementary Table 1. Panels B and C are shown without error bars to make the curves shown easier to see; these panels are included with error bars in the Supplementary Material.

There were no intergroup differences in average body weight at the start of the dietary interventions or at the start of chemotherapy. Docetaxel treatment resulted in a rapid decrease in body weight by an average of 14%, but weight loss did not differ across the diet groups (Supplemental Table 1). Body weight in all docetaxel-treated groups had recovered by approximately day 60 (Fig. 6). As expected, tumor growth was delayed by docetaxel treatment (Fig. 6). All tumors experienced a lag in growth following docetaxel treatment that lasted approximately 10 days. None of the tumors exhibited a complete regression in volume in response to taxane treatment. There was no growth delay of tumors in mice treated with docetaxel vehicle, which reached an average volume of 3,000 mm^3^ at 54.4 days, whereas tumors in docetaxel treated mice took 63.4 (control diet), 63.9 (low dose ellagic acid diet) and 61.9 (high dose ellagic acid diet) days to reach the same volume (Supplemental Table 1). Thus, dietary ellagic acid had no impact on the effectiveness of docetaxel as we did not observe significant differences in tumor growth rates, tumor growth delay, or overall survival in groups treated with docetaxel and fed the low or high dose ellagic acid diets compared to the group treated with docetaxel and fed control diet (Fig. 6 and Supplemental Table 1).

## Discussion

The major findings of this study are that ellagic acid has the potential to adversely affect taxane treatment of CRPC by reducing their tubulin polymerizing effect, but by itself caused modest tubulin polymerization and bound to tubulin albeit with considerably less affinity than taxane drugs. In unpublished preliminary experiments, we also found that ellagic acid increased expression of MAP2, a protein involved in assembly and stability of microtubules^28^, suggesting that cellular effects of ellagic acid may be mediated by upregulation of proteins involved in microtubule dynamics. While no *in vivo* evidence for this interaction was obtained and ellagic acid did not alter the *in vitro* cytotoxicity of taxanes, the *in vitro* effects of ellagic acid on the mechanism by which docetaxel and cabazitaxel inhibit growth of prostate cancer cells are some additional cause of concern. On the other hand, ellagic acid inhibited P-glycoprotein-mediated drug efflux, which would be consistent with enhancement of taxane effects. These observations all indicate the potential of ellagic acid to influence treatment of CRPC with taxane drugs in a variety of ways. This is significant because a range of plants that are found in human foods and supplements contain this strong antioxidant compound and it has been pursued for development as a cancer preventive agent^8^. Our findings indicate that caution is warranted in the use of ellagic acid and ellagic acid-rich foods and supplements by (prostate) cancer patients while on taxane therapy. On the other hand, ellagic acid may have some promise as an agent with some tubulin polymerizing activity that is extremely well tolerated.

Effects of BRB constituents on microtubule dynamics have not been investigated previously, but it has been reported that an anthocyanin-containing bilberry extract can cause depolymerization of microtubules in MCF-7 breast cancer cells^29^. Furthermore, other natural polyphenolic compounds with structural similarity to ellagic acid have been reported to interact with microtubules and disrupt their dynamics^30–32^. Although we observed some inhibition of microtubule assembly by BRB in a cell-free assay, this result is likely an experimental artifact and not biologically relevant since the same BRB extract did not elicit any effects on the morphology of microtubules in cells. In our experiment, the effects of BRB extract and PCA on tubulin polymerization were evaluated by combining them with a solution containing purified tubulin protein, GTP, and DAPI, then measuring the increase in fluorescence intensity as an indicator of polymerization. The simplicity of this assay allows for straightforward identification of compounds that directly interact with tubulin, but lacks the complexity of the intracellular environment. Given that our BRB extract is a mixture of numerous constituents, it is possible that one or more of the components interfere with tubulin protein assembly or GTP hydrolysis in this assay, but do not affect microtubule dynamics under physiological conditions. The absence of effects by BRBs on the morphology of microtubules observed in 22Rv1 cells supports this notion.

Ellagic acid has also been shown to lower androgen receptor expression in androgen receptor-positive LNCaP cells (which are androgen dependent)^33^ and we have found the same effect in 22Rv1 CRPC cells (which are androgen-sensitive) and observed reduced protein expression of AR splice variants (unpublished data). However, it is not clear whether this ellagic acid effect signifies a benefit because in the present study 22Rv1 cells were *in vitro* growth inhibited by ellagic acid to the same extent as PC-3 cells, which do not express androgen receptor. Nonetheless, these findings suggest the possibility that ellagic acid may also interfere with androgen receptor-targeted therapies, which merits further research. In addition, there is a report indicating that different androgen receptor splice variants may differentially affect taxane chemotherapy efficacy^34^.

That BRB extract does not appear to have much effect on *in vitro* taxane drug efficacy or tubulin polymerization should not come as a surprise, given the fact that *in vivo* effects of oral treatment are dependent on intestinal metabolism and absorption as well as on systemic metabolism, e.g., in the liver. Bioavailable levels of many BRB compounds will probably not reach sufficient circulating levels to have an effect on taxane mechanisms of action, uptake, or metabolism at target tissues. However, the anthocyanin metabolite PCA, which does reach the prostate of rats fed BRBs (unpublished data), did not have much influence on taxane cytotoxicity of CRPC cells or tubulin polymerization. It is possible that the inability of PCA to elicit a response in our assays is due to the concentrations used in this study. We did not investigate concentrations of PCA higher than 10 μg/mL, thus it remains possible that higher pharmacological doses of PCA have activity. Bioavailability of orally administered ellagic acid has been reported to be poor in rats^35^, but other reports indicate that in humans it is absorbed and bioavailable after oral administration of black raspberries and pomegranate juice^36,37^. Furthermore, *in vivo* inhibitory effects of oral ellagic acid administration have been reported on induction of mammary gland tumors in rats^11,12^ and lung cancer in mice^38^, as well as on growth of bladder cancer cells in nude mice^39^. These data indicate uptake and effects of ellagic acid on internal tissues in addition to its direct effects on the gastrointestinal tract^8^.

Circulating and intracellular levels of taxanes are regulated by several factors including metabolism by CYP3A4 and efflux activity of ABC transporters. The inhibition of efflux activity by ellagic acid we observed has also been reported by others who additionally found inhibition by anthocyanin aglycones^40^. In the present study, BRB extract at low concentrations also inhibited efflux activity in 22Rv1 cells, but PCA did not. Anthocyanins inhibited CYP3A4 activity in human hepatic microsomes, but only at high concentrations (50–100 μM)^41^. We found no effects of ellagic acid or PCA on CYP3A4 activity in microsomes, but BRB extract inhibited activity at high concentrations (100–1,000 μg/mL). Although we did not study the effects of individual anthocyanins in these assays, our results with BRB extract appear to be consistent with findings of others demonstrating that effects of anthocyanins on efflux transporters and CYP3A4 activity is associated with anthocyanin aglycones rather than glycoside conjugates that are present in BRB^40–42^.

In conclusion, we did not find evidence to support our hypothesis that BRB can enhance the efficacy of taxane chemotherapy. Neither BRB extract nor the anthocyanin metabolite PCA enhanced effects of taxane chemotherapeutics. Likewise, we did not find any effect of BRB or PCA on taxane metabolism or mechanisms of resistance. Ellagic acid, on the other hand, did have an inhibitory effect on P-glycoprotein-mediated drug efflux, which would be consistent with enhancement of taxane effects. In addition, we observed binding of ellagic acid to tubulin and a novel microtubule polymerizing effect of ellagic acid *in vitro* and we obtained evidence that it may interfere with microtubule polymerization induced by taxane drugs potentially reducing their effectiveness. However, we did not observe a reduction of *in vitro* taxane cytotoxicity by dietary ellagic acid and there was no effect on docetaxel efficacy on growth of 22Rv1 tumors in nude mice. Taken together our findings suggest that while consumption of BRB during taxane chemotherapy will not provide a therapeutic benefit, it is safe and not likely to reduce drug effectiveness or increase toxicity. However, some of our results indicate a potential adverse interaction between taxane chemotherapy and ellagic acid alone. The mechanism of action of taxanes, microtubule stabilization, is not associated with oxidative stress, but many other agents – such as platinum-based chemotherapeutic drugs and alkylating agents – induce apoptosis primarily by generating reactive oxygen species and producing high levels of oxidative stress. BRB anthocyanins and ellagic acid are known to exert potent antioxidant activity^43^ and it is possible that even low levels could antagonize prooxidant activities of such drugs. Thus, further research is warranted particularly regarding our observations indicating the potential of ellagic acid to influence treatment of CRPC with taxane drugs in a variety of ways that have uncertain but potentially hazardous clinical implications.

## Materials and Methods

### Reagents and chemicals

Docetaxel and sulforhodamine B were purchased from Sigma-Aldrich (St. Louis, MO). Cabazitaxel (Sanofi, Bridgewater, NJ) was a generous gift from Dr. Robert Nagourny (Rational Therapeutics, Long Beach, CA). Ellagic acid was obtained from Indofine Chemical (Hillsborough, NJ). Urolithin A was obtained from Santa Cruz (Dallas, TX). Protocatechuic acid was purchased from LKT Labs (St. Paul, MN). Purified tubulin protein was purchased from Cytoskeleton (Denver, CO). Phenol-red free matrigel was obtained from Corning (Bedford, MA). DMSO, ethanol, and trichloroacetic acid were obtained from Thermo Fisher Scientific (Waltham, MA). Antibodies against β-actin (catalog #3700), β-tubulin (#2128), anti-mouse Alexa Fluor 488 (#4408), and anti-rabbit Alexa Fluor 555 (#4413) were obtained from Cell Signaling Technologies (Danvers, MA). GAPDH (#365062) antibody was obtained from Santa Cruz Biotechnology (Dallas, TX). IRDye 800 anti-rabbit (#926-32211) and IRDye 680 anti-mouse (#926-68070) antibodies were obtained from Li-Cor Biotechnology (Lincoln, NE).

### Preparation of black raspberry extract

An ethanol/H_2_O (80/20) soluble BRB extract was prepared from a single batch of lyophilized black raspberry powder (Berri Products, Corbett, OR) which was stored at −20 °C. 200 g BRB powder was combined with 80% ethanol and stirred overnight at room temperature. The resulting mixture was vacuum-filtered. The solvents were removed to the extent possible by rotary evaporation at reduced pressure, yielding a syrup. The remaining solvent was removed under a nitrogen stream. Final weights of extracts were obtained and BRB extract were reconstituted in 100% ethanol and stored at −20 °C.

### Cell lines and cell culture

22Rv1 and PC-3 cells were obtained from American Type Culture Collection (Manassas, VA) and C4-2 cells were a generous gift from Dr. Leland Chung (Cedars Sinai Medical Center). Cells were used for experiments within 20 passages from arrival and screened for mycoplasma contamination using a MycoAlert™ PLUS mycoplasma detection kit (Lonza Walkersville, Walkersville, MD). All cells were cultured in RPMI 1640 (Life Technologies, Grand Island, NY) supplemented with 10% FBS, 100 IU/mL penicillin, and 100 μg/mL streptomycin. Cells were maintained at 37 °C in a humidified 5% CO2 incubator. Twenty four hours prior to experiments, media was replaced with phenol-red free RPMI or DMEM containing 10% dextran charcoal stripped FBS, 100 IU/mL penicillin, and 100 μg/mL streptomycin.

### Proliferation assays

The sulforhodamine B (SRB) assay was performed to evaluate growth. Cells were seeded on 96-well plates at a density of 2,000 cells per well 24 hours prior to the addition of test compounds to culture media. Cells were incubated for 72 hours, and then fixed with 1% trichloroacetic acid and stained with 0.057% SRB. Wells were washed with 1% acetic acid and the remaining dye was solubilized in 10 mM Tris. Optical density at 510 nm was measured with the Synergy HTX plate reader (Biotek, Highland Park, VT). Incorporation of 5-ethynyl-2′-deoxyuridine (EdU) into DNA was measured to assess cell proliferation using the Click-iT EdU Microplate Assay (Invitrogen). Cells were labeled with EdU for 2 hours then fixed. Cells were treated with buffer containing CuSO4 and Oregon Green 488 azide for 25 min, incubated with 5% BSA for 30 min, and then incubated with anti-Oregon Green antibody conjugated to horseradish peroxidase for 30 minutes, followed by incubation with Amplex Red for 15 minutes. Fluorescence intensity (excitation: 560 nm, emission: 590 nm) was measured with a SpectraMax M5 spectrophotometer (Molecular Devices, Sunnyvale, CA). Results are expressed as percent proliferation relative to vehicle treated control cells. Each treatment condition was replicated 3 times in a single experiment, which was repeated at least 3 times.

### Surface plasmon resonance

Binding affinity of compounds to tubulin was evaluated by surface plasmon resonance using a Biacore T-2000 instrument (GE Life Sciences, Uppsala, Sweden). Purified tubulin protein (100 μg/mL in 10 mM sodium acetate pH 4.0) was immobilized on CM5 sensor chips (GE Life Sciences) by amine coupling using N-hydroxysuccinimide (NHS) and N-ethyl-N-(dimethyl-aminopropyl)-carbodiimide (EDC) using filtered (0.2 μm) PBS-P + buffer (GE Life Sciences). A minimum of 9,000 response units (RU) were immobilized to the chip. For each experiment, 1 flow channel of the CM5 chip was prepared in the absence of tubulin for reference subtraction. The running buffer (10 mM HEPES pH 7.4, 150 mM NaCl, 3.4 mM EDTA, 0.05% (vol/vol) Tween-20) was supplemented with 2% DMSO. Test compounds (serially diluted, 0–200 μM) were injected at a flow rate of 30 μL/min. Surface plasmon resonance data are representative of duplicate injections acquired for three independent experiments. The equilibrium dissociation constants (KD) were calculated from steady-state affinity after DMSO solvent correction.

### Tubulin polymerization assay

Effects of compounds on tubulin polymerization were evaluated by monitoring incorporation of a fluorescence-based reporter (Cytoskeleton, Denver, CO) into microtubules as polymerization occurs. Purified tubulin (2 mg/mL) was suspended in 80 mM PIPES pH 6.9, 2.0 mM MgCl2, 0.5 nM EDTA, 1.0 GTP and 15% glycerol, and experimental compounds or vehicle controls were added. Fluorescence intensity (excitation: 360 nm, emission: 420 nm) was measured at 1 minute intervals for 60 minutes using a Synergy HTX plate reader (Biotek).

### Efflux Assay

Effects on P-glycoprotein activity were determined by measuring efflux of calcein AM, the fluorescent substrate of P-glycoprotein. Cells were plated in 96 well plates and cultured to 80% confluence, then incubated at 37 °C with compounds in phenol-red free culture media. After 24 hours, cells were treated with cold media containing 0.25 μM calcein AM and incubated for 1 hour at 4 °C to facilitate uptake. Cells were collected, washed with PBS, resuspended in phenol-red free culture media and incubated at 37 °C. Cell culture media was collected at 0, 2 and 4 hours, and calcein fluorescence intensity (excitation: 488 nm, emission 530 nm) of samples was measured using a Synergy HTX plate reader (Biotek).

### CYP3A4 Activity

Effects on CYP3A4 activity were evaluated using insect microsomes expressing human CYP3A4 enzyme and the Vivid CYP3A4 Green Screening Kit (Life Technologies). Microsomes were incubated with glucose-6-phosphate, glucose-6-phosphate dehydrogenase, NADP+, and DBOMF (non-fluorescent CYP3A4 substrate, which releases a fluorescent compound when metabolized by CYP3A4) in presence of test compounds or control vehicle. Fluorescence (excitation: 490 nm, emission: 520 nm) was measured using a Synergy HTX plate reader (Biotek).

### Confocal microscopy

22Rv1 cells were plated on Lab-Tek 4-well chamber slides (Nunc, Naperville, IL) and cultured to approximately 80% confluency. Cells were treated with compounds for 24 hours, fixed with 3.7% formaldehyde, washed with PBS, permeabilized for 10 minutes with 0.1% Triton-X 100, and blocked with 5% BSA for 30 minutes. Slides were then incubated overnight at 4 °C with primary antibodies, followed by incubation with fluorescent secondary antibodies for 2 hours at room temperature in a dark, humidified chamber. Cover slips were mounted on slides with ProLong Gold Antifade Mountant containing DAPI (Life Technologies). Fluorescent images were acquired using a Zeiss LSM 710 confocal laser-scanning microscope using 40x or 63x objectives. Acquired images were enhanced for brightness and contrast.

### Western blot analysis

Quantification of cellular levels of polymerized and soluble tubulin was performed by western blotting. Total protein was isolated from cells using radioimmunoprecipitation assay (RIPA) buffer (Sigma, St. Louis, MO) followed by centrifugation ot separate the soluble (monomeric) tubulin fraction from the insoluble polymerized fraction. Protein concentration was quantified using the bicinchoninic acid method with the Piece BCA Protein Assay Kit (Thermo Fisher). Soluble and polymerized protein lysates were loaded onto 4–12% NuPAGE Bis-Tris gels (Invitrogen, Thermo Fisher), resolved by electrophoresis, and then transferred to PVDF membranes. Membranes were incubated with blocking buffer (5% BSA or 5% non-fat milk) for 30 minutes, and then probed with primary antibody against β-tubulin (Cell Signaling) overnight at 4 °C. Membranes were incubated with IRDye infrared fluorescent secondary antibodies (Li-Cor) and bands were detected and quantified using the Odyssey CLx Infrared Imaging System (Li-Cor).

### Prostate cancer xenografts in nude mice

Animal care and experiments were approved by the Animal Care Committee (ACC) at the University of Illinois at Chicago and in compliance with the U.S. Public Health Service Policy on Humane Care and Use of Laboratory Animals and with the ARRIVE Guidelines. The *in vivo* effects of dietary consumption of ellagic acid on the efficacy of docetaxel were studies in a subcutaneous xenograft model with 22Rv1 cells. Male six-week-old athymic nude mice (Hsd:Athymic Nude-*Foxn1nu*) were obtained from Envigo (Indianapolis, IN). 22Rv1 cells were suspended in matrigel solution (4 mg/mL phenol-red free matrigel obtained from Corning in PBS) and injected subcutaneously on both flanks, 1 × 10^6^ cells in 200 μL per injection, while mice were anesthetized with ketamine (100 mg/kg) and xylazine (5 mg/kg). Mice were fed Teklad irradiated 7912 natural ingredient diet (Envigo) until tumors reached approximately 500 mm^3^, then were randomized to one of the following diets: irradiated AIN-93M control diet (Envigo) or AIN-93M diet containing 2 or 4 g/kg ellagic acid (low and high ellagic acid group, respectively). Ellagic acid was sterily mixed in a premix of sucrose that was subsequently mixed under sterile conditions into the diet that contained less sucrose to accommodate the premix; the diets were stored at −20 °C until used. One week after start of these diets, mice received docetaxel (15 mg/kg; 0.4 mL injection volume) or vehicle injections, administered intraperitoneally three times at 3-day intervals. Docetaxel solution was prepared by diluting stock solution (22.5 mg/mL in 100% EtOH) in diluent (1 volume polysorbate in 18 volumes 5% glucose in sterile water) to a final concentration of 1.125 mg/mL. Vehicle injection solutions were prepared similarly, except docetaxel was replaced with 100% EtOH. Tumor size was measured three times per week using calipers until tumor volume exceeded 3,000 mm^3^ at which point the animals were euthanized. Six animals were used per diet/treatment group except for 10 mice in the comparison group given control diet but no docetaxel. No a priori sample size calculation was performed because tumor take and response to docetaxel were not known, but the number of animals was based on results of published studies^44^.

### Data analysis

All *in vitro* experiments were performed at least 3 times. Results are expressed as mean ± standard deviation (SD), unless otherwise specified. Comparisons were performed using Student’s t test or one way analysis of variance (ANOVA) followed by a post-hoc test when appropriate. Statistical analysis was performed with GraphPad. A p value < 0.05 was considered significant. Primary endpoints of the *in vivo* xenograft study were xenograft tumor volume, calculated using the following formula: length × (2 × width) × 0.523, and tumor growth delay, calculated with the following formula: (time for treated group to reach tumor volume of 3,000 mm^3^)−(time for control group to reach tumor vol. of 3,000 mm^3^)^44^. Outliers were identified using the extreme studentized deviate test (Grubbs’ test)^45^ and excluded from data analysis.

### Note

Many of the methods used in this study were identical to those used by us as previously reported^46^.

## Supplementary information

Supplementary Material
