# Supplementary material for: Effects of Black Raspberries and Their Ellagic Acid and Anthocyanin Constituents on Taxane Chemotherapy of Castration-Resistant Prostate Cancer Cells
Source: Sci Rep. 2019 Mar 13;9:4367. doi: 10.1038/s41598-019-39589-1 (PMC6416359; doi:10.1038/s41598-019-39589-1)

SUPPLEMENTARY MATERIAL

Effects of Black Raspberries and Their Ellagic Acid and Anthocyanin Constituents on Taxane Chemotherapy of Castration-Resistant Prostate Cancer Cells

Jillian N. Eskra, Michael J. Schlicht & Maarten C. Bosland

Supplementary Figure 1

Effects of 1-30 μM ellagic acid on tubulin polymerization in 22Rv1 cells measured by levels of soluble and polymerized determined by western blot (**A**); effects on tubulin polymerization in 22Rv1 cells of 10 nM cabazitaxel (CBZ) alone or in combination with 1 mg/ml black raspberry extract (BRB) with band intensity was quantified using Li-Cor Odyssey software (**B**), or 10 μM ellagic acid (EA) (**C**).

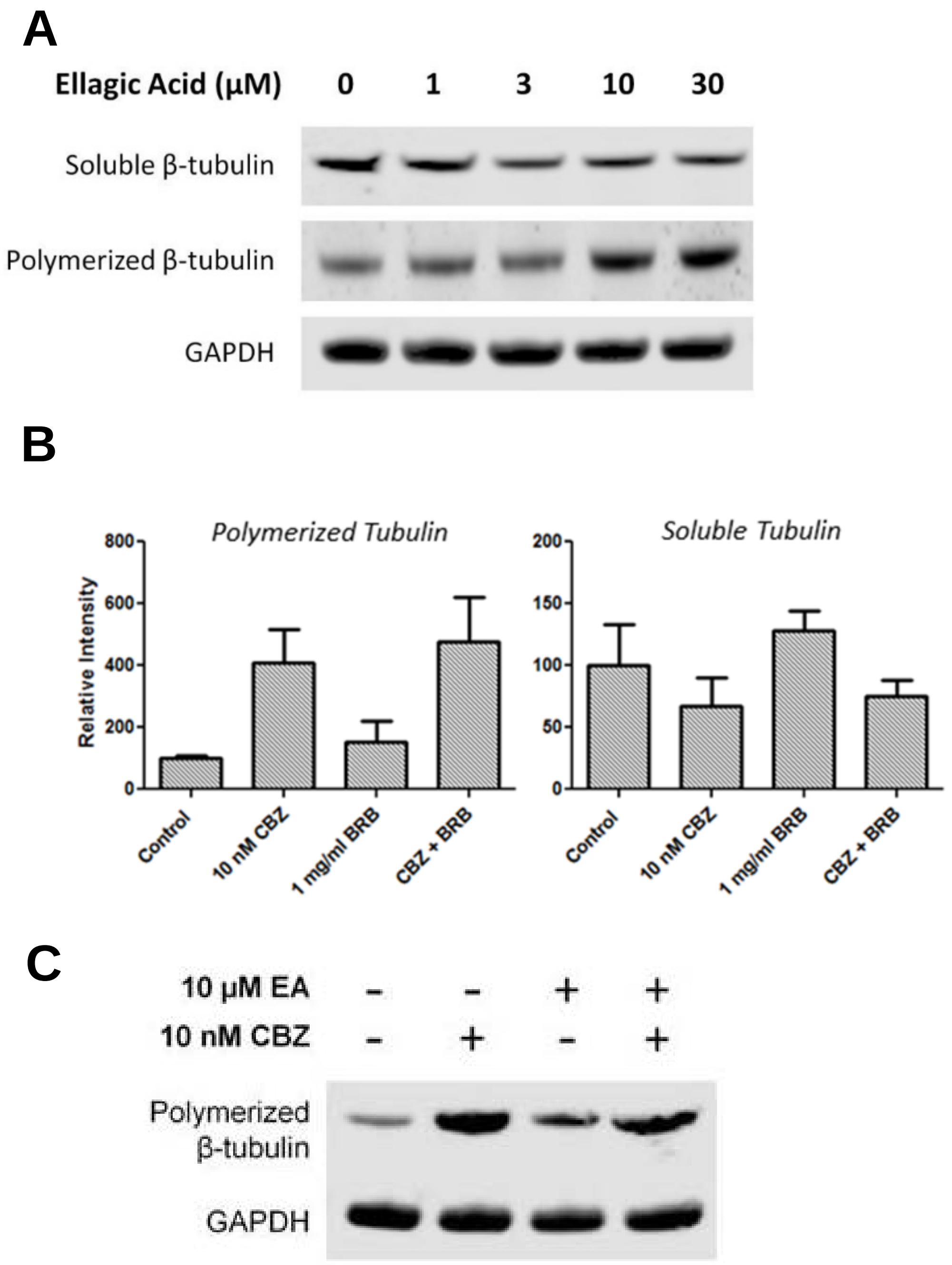

Supplementary Table 1

Effects of dietary ellagic acid on efficacy of docetaxel on 22Rv1 xenografts

	Vehicle + Ctrl Diet	DX + Ctrl Diet	DX + Low EA	DX + High EA
**Dietary EA (g/kg diet)**	0	0	2	4
**Treatment [a]**	Vehicle	DX	DX	DX
**Number of animals**	6	10	6	6
**No. of measureable tumors at day 35 (total 22Rv1 injections)**	9 (12)	16 (20)	7 (12)	8 (12)
**No. of tumors included in data analysis**	6	10	5	6
**No. of tumors excluded from data analysis**				
Tumors < 350 mm^3 at day 35	3	5	1	1
Tumors > 2500 mm^3 at day 35	0	1	1	1
**Average body weight (g ± SD)**				
At start of dietary intervention (day 28)	31.0 ± 1.7	31.2 ± 3.5	28.3 ± 4.5	29.8 ± 0.8
At start of therapy (day 35)	31.2 ± 1.2	30.9 ± 2.4	29.3 ± 3.9	30.0 ± 0.9
One week after therapy (day 49)	32.2 ± 1.2	26.8 ± 3.6 [b]	24.5 ± 3.3 [b]	26.5 ± 2.1 [b]
**Average tumor volume (mm^3 ± SD)**				
At start of dietary intervention (day 28)	454.2 ± 231.3	477.2 ± 256.6	405.0 ± 136.0	408.8 ± 200.7
At start of therapy (day 35)	748.3 ± 262.9	1166.9 ± 489.2	762.0 ± 155.9	1301.8 ± 918.4
One week after therapy (day 49)	1972.0 ± 640.1	1730.8 ± 921.2	1584.2 ± 484.0	1591.8 ± 808.4
**Time to reach 3,000 mm^3 (average number of days ± SD)**	54.4 ± 5.7	63.4 ± 5.4 [b]	63.9 ± 5.5 [b]	61.9 ± 3.0 [b]
**Average tumor growth delay (days)**	Reference	9.0	9.5	7.5

[a] Vehicle (saline) and DX (15 mg/kg body weight) treatments were administered by IP injection on days 35, 39, and 43.
[b] $p < 0.05$ for difference with the vehicle + control diet group (ANOVA)
DX = Docetaxel; EA = Ellagic Acid; Ctrl = Control; SD = Standard Deviation.

Original images of bands in Supplementary Figures 1 and 2 as detected using an Odyssey CLx Infrared Imaging System

Black & white images **Fluorescence images**

<u>Supplementary Figure 1A</u>

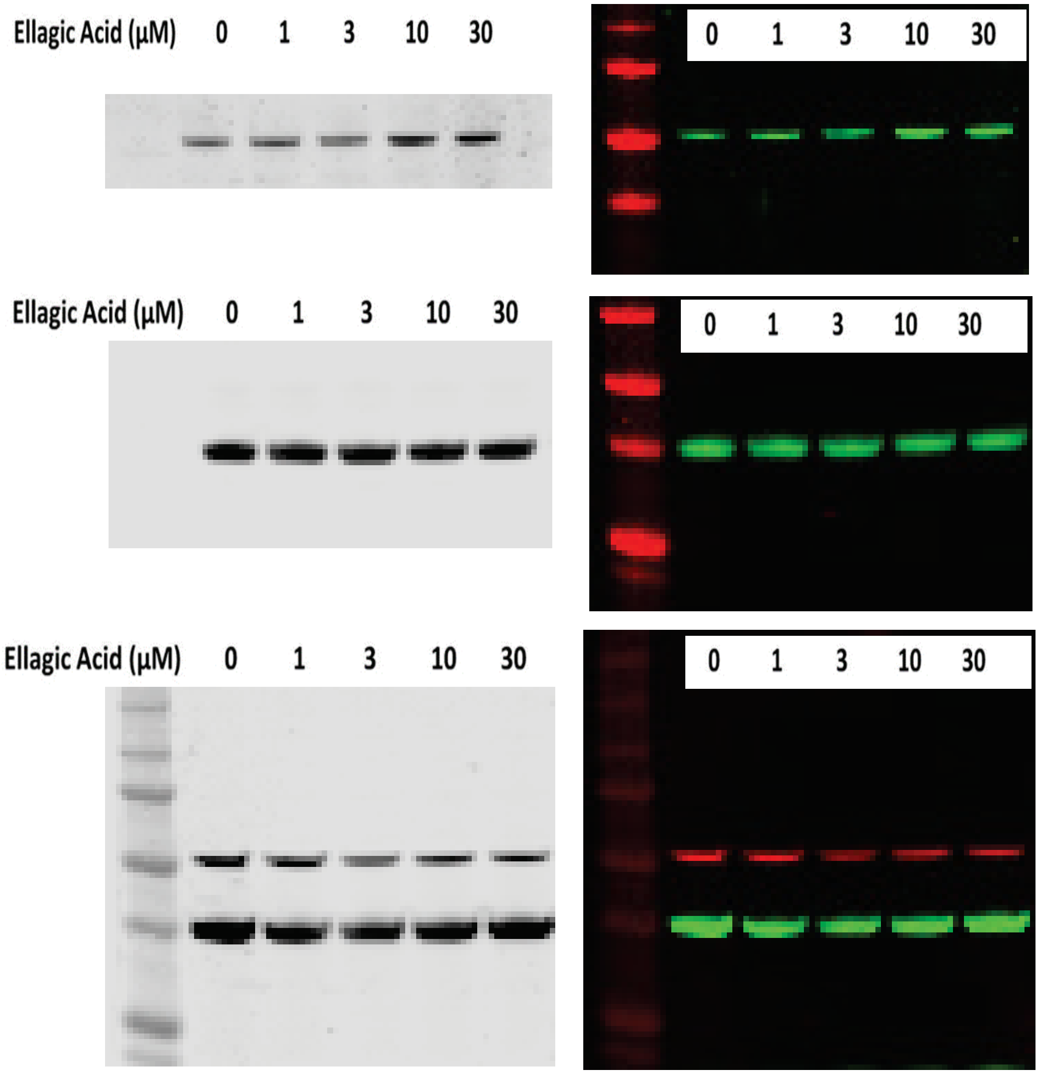

← Polymerized β-tubulin (50 kDa)

← GADPH (36 kDa)

← Soluble β-tubulin (50 kDa)

← GADPH (36 kDa)

<u>Supplementary Figure 1C</u>

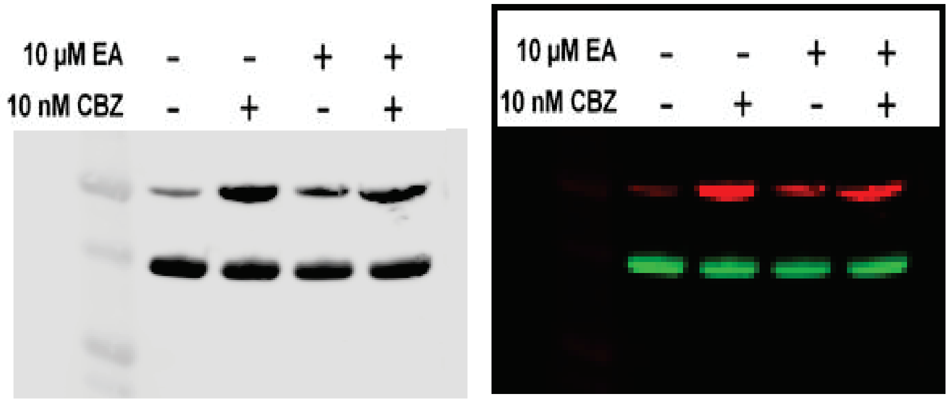

← Polymerized β-tubulin (50 kDa)

← GADPH (36 kDa)

Panels A and B of Figure 3 with error bars showing

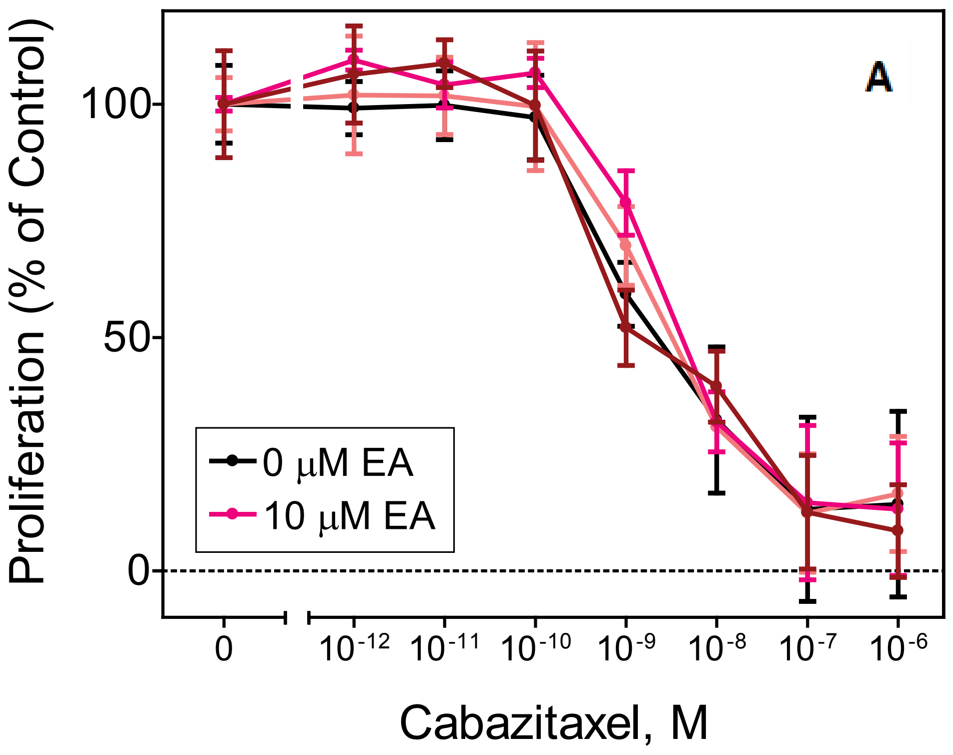

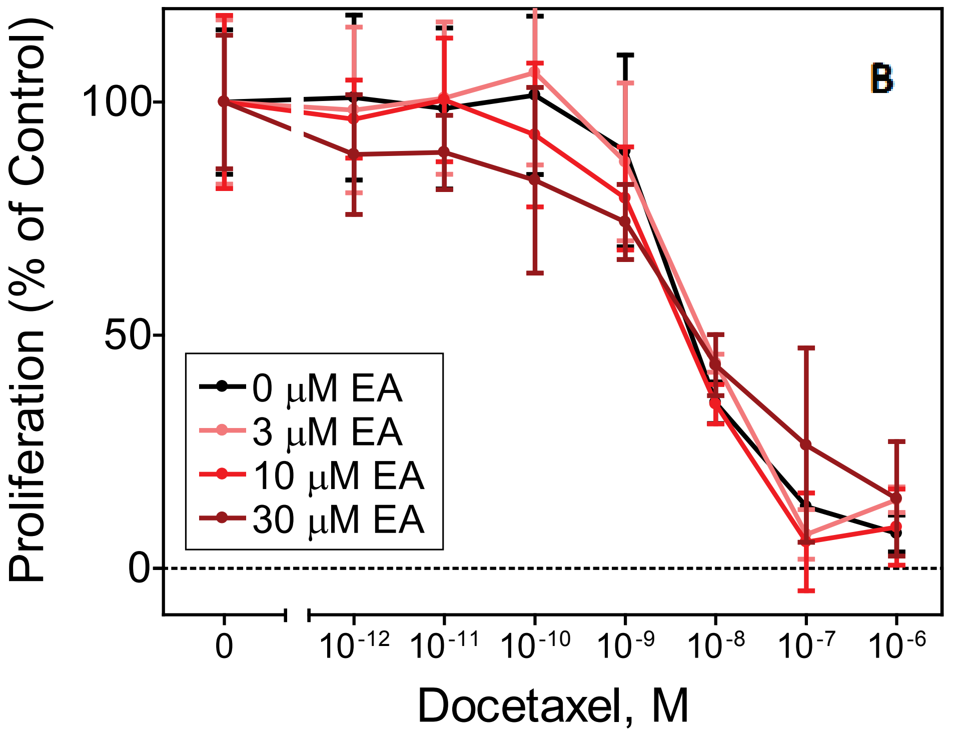

Panel B of Figure 6 of mean body weights with standard deviation error bars showing

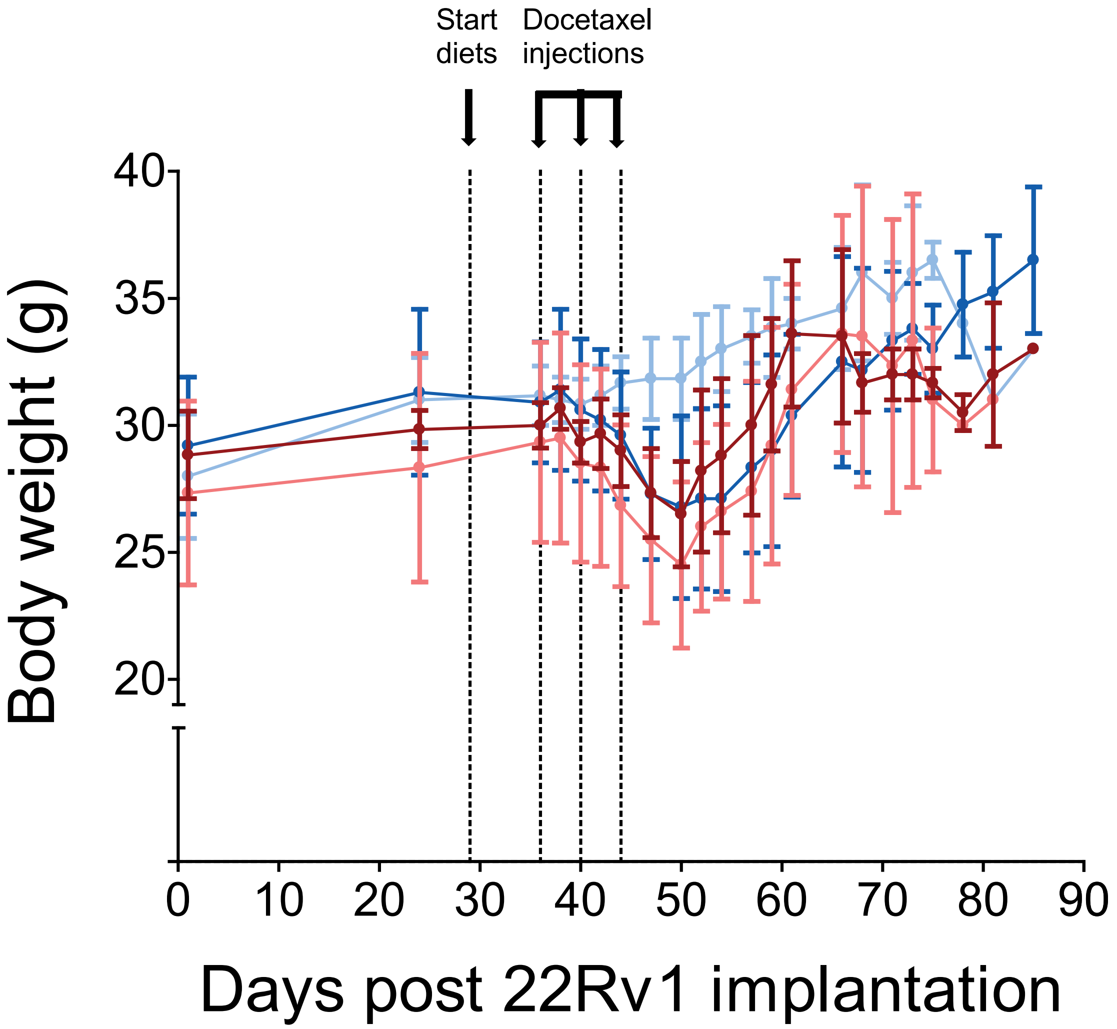

Panel C of Figure 6 of mean tumor volumes with standard deviation with error bars showing

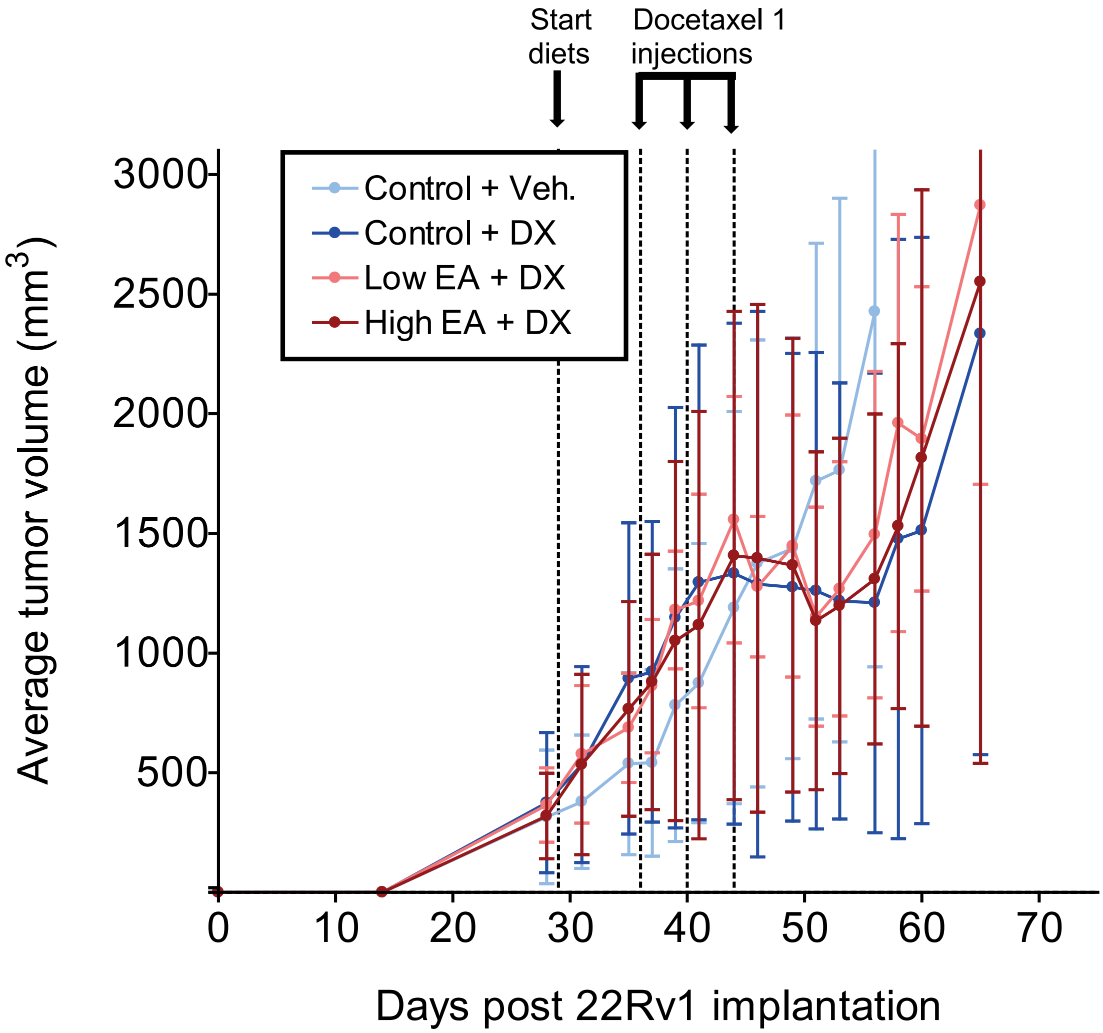

Supplement: Supplementary file 1 — Supplementary Material [file 41598_2019_39589_MOESM1_ESM.pdf]
